# Evaluating accountability, transparency, and bias in AI-assisted healthcare decision- making: a qualitative study of healthcare professionals’ perspectives in the UK

**DOI:** 10.1186/s12910-025-01243-z

**Published:** 2025-07-08

**Authors:** Saoudi CE Nouis, Victoria Uren, Srushti Jariwala

**Affiliations:** 1 Biochemistry Department, Worcester Royal Hospital, Charles Hastings Way, Worcester, WR5 1DD UK; 2 Master of Business Management, Aston University, Birmingham, UK; 3 Aston Business School, Aston University, Birmingham, UK; 4 Microbiology Department, Worcester Royal Hospital, Worcester, UK

**Keywords:** Electronic health record, Artificial intelligence, Clinical Decision-Making, Accountability, Transparency, Bias, Qualitative research, Healthcare ethics

## Abstract

**Background:**

While artificial intelligence (AI) has emerged as a powerful tool for enhancing diagnostic accuracy and streamlining workflows, key ethical questions remain insufficiently explored—particularly around accountability, transparency, and bias. These challenges become especially critical in domains such as pathology and blood sciences, where opaque AI algorithms and non-representative datasets can impact clinical outcomes. The present work focuses on a single NHS context and does not claim broader generalization.

**Methods:**

We conducted a local qualitative study across multiple healthcare facilities in a single NHS Trust in the West Midlands, United Kingdom, to investigate healthcare professionals’ experiences and perceptions of AI-assisted decision-making. Forty participants—including clinicians, healthcare administrators, and AI developers—took part in semi-structured interviews or focus groups. Transcribed data were analyzed using Braun and Clarke’s thematic analysis framework, allowing us to identify core themes relating to the benefits of AI, ethical challenges, and potential mitigation strategies.

**Results:**

Participants reported notable gains in diagnostic efficiency and resource allocation, underscoring AI’s potential to reduce turnaround times for routine tests and enhance detection of abnormalities. Nevertheless, accountability surfaced as a pervasive concern: while clinicians felt ultimately liable for patient outcomes, they also relied on AI-generated insights, prompting questions about liability if systems malfunctioned. Transparency emerged as another major theme, with clinicians emphasizing the difficulty of trusting “black box” models that lack clear rationale or interpretability—particularly for rare or complex cases. Bias was repeatedly cited, especially when algorithms underperformed in minority patient groups or in identifying atypical presentations. These issues raised doubts about the fairness and reliability of AIassisted diagnoses.

**Conclusions:**

Although AI demonstrates promise for improving efficiency and patient care, unresolved ethical complexities around accountability, transparency, and bias may erode stakeholder confidence and compromise patient safety. Participants called for clearer regulatory frameworks, inclusive training datasets, and stronger clinician–developer collaboration. Future research should incorporate patient perspectives, investigate long-term impacts of AI-driven clinical decisions, and refine ethical guidelines to ensure equitable, responsible AI deployment.

**Trial registration:**

: Not applicable.

**Supplementary Information:**

The online version contains supplementary material available at 10.1186/s12910-025-01243-z.

## Introduction

Healthcare systems worldwide have seen a rapid upsurge in the integration of electronic health record (EHR) systems, with adoption rates exceeding 90% in many countries [1]. In places like the United States, the rate is as high as 96%, which has led to the generation of vast repositories of structured and unstructured healthcare data [1, 2]. Structured elements, such as diagnoses and procedures, are often combined with clinical narratives, resulting in extensive data warehouses that have become pivotal for both biomedical research and evidence-based practice [3]. Leveraging these comprehensive datasets, artificial intelligence (AI) has gained traction as a means to improve clinical decision-making and to streamline workflows in hospitals and clinics. By analyzing historical and real-time clinical data, AI-driven systems can identify meaningful patterns, facilitate accurate diagnoses, recommend targeted treatment options, and assist in designing personalized care plans [4, 5]. These capabilities underscore AI’s potential to revolutionize healthcare, offering hope for enhanced patient outcomes, better operational efficiency, and reduced healthcare spending.

Early AI systems in healthcare were largely rule-based, utilizing “if-then” structures to handle routine tasks. Over time, AI expanded to include Machine Learning (ML), Deep Learning (DL), symbolic AI, and other approaches that aim to mimic human cognition [6]. Research has demonstrated the effectiveness of these technologies in managing large datasets and tackling complex healthcare challenges, such as accurately detecting diabetic retinopathy from retinal images [7], identifying mitosis in breast cancer histology [8], and predicting cardiovascular risk [9]. In addition, AI-based tools have been used to identify individuals at heightened risk for suicide attempts or those who might soon require palliative care interventions [10]. These examples highlight the breadth of AI’s impact, indicating that it can deliver improvements in diagnosis, prognosis, and the execution of individualized treatment strategies.

Beyond diagnostics and prognostics, AI also promises significant economic benefits. In the United States, up to 18% of the GDP is spent on healthcare, a substantial fraction of which is attributed to avoidable inefficiencies and unnecessary services [11]. By using real-time data analytics, predictive modeling, and automation, AI can curtail overtreatment, minimize human errors, and optimize resource use, thereby boosting cost-effectiveness [12]. A variety of studies—ranging from retrospective analyses in radiology to large-scale investigations of hospital workflows—have documented time savings, reductions in overhead, and fewer readmissions when AI systems supplement or replace traditional processes [13, 14]. Notably, AI can automate repetitive tasks such as scheduling, lab result triaging, and routine charting, freeing clinicians to focus on complex decision-making and patient engagement [15]. Collectively, these advantages can enable better patient flow, reduced waiting times, and a more robust return on investment for healthcare institutions willing to adopt AI-based tools.

Despite these encouraging developments, several key ethical concerns remain underexamined.

Chief among them are accountability, transparency, and bias in AI-assisted decision-making. The “black box” nature of deep neural networks and other advanced algorithms can make it nearly impossible for clinicians to interpret how the system arrives at specific recommendations [16]. When a healthcare outcome is suboptimal or harmful, it can be difficult to attribute responsibility—should it lie with the engineer who designed the AI, the institution that approved its use, or the clinician who ultimately acted upon AI-generated insights [17]? While some argue that clinicians must retain ultimate responsibility for patient care, others suggest that developers and regulatory bodies share moral and legal accountability when the internal logic of AI systems is inscrutable [18]. Such ambiguity becomes especially problematic when issues of bias arise, wherein AI models systematically underperform for underrepresented patient groups or inadvertently aggravate existing health disparities [19]. Sources of bias can stem from skewed training data, flawed algorithmic design, or real-time mismatches between model assumptions and patient populations [20]. Indeed, machine learning algorithms often learn patterns that reflect societal or institutional biases, potentially introducing unequal treatment or misdiagnoses in vulnerable communities.

Although scholars have started to address these topics theoretically, qualitative research capturing the practical, day-to-day experiences of healthcare professionals who rely on AI is comparatively scarce [21]. Most existing studies documenting AI’s efficacy or shortcomings rely on retrospective or quantitative metrics—such as diagnostic accuracy or cost savings— that overlook the nuanced perspectives of clinicians, nurses, and healthcare administrators grappling with AI systems in real time [22, 23]. Understanding these frontline viewpoints is critical, as it is often the clinical staff who must reconcile AI recommendations with patient preferences, clinical guidelines, and broader ethical obligations. Moreover, frontline users are frequently the first to notice system limitations, from algorithmic “blind spots” to workflow incompatibilities. Without their insights, the path toward ethically sound and practically feasible AI integration remains incomplete.

While a growing body of research explores AI’s diagnostic accuracy and cost-effectiveness in healthcare, comparatively little work has investigated how healthcare professionals experience and negotiate the ethical challenges of AI-assisted decision-making. Existing quantitative studies often measure algorithmic performance but do not capture the nuanced, frontline perspectives on accountability for clinical outcomes, the transparency of ‘black box’ algorithms, or the potential for systemic biases in patient care. Without these qualitative insights, policymakers and clinical leadership risk implementing AI-driven tools that may overlook the day-to-day ethical dilemmas faced by clinicians, potentially eroding patient trust and quality of care. By focusing on the lived experiences of healthcare professionals, this qualitative study aims to address that gap, offering actionable findings that can inform regulatory guidelines, institutional policies, and targeted training programs for ethical and effective AI integration.

Through a qualitative approach—employing in-depth interviews and thematic analysis—we seek to explore how clinicians incorporate AI outputs into patient care, how they perceive the “black box” phenomenon, and how they address or respond to potential biases. By capturing these insights, the study can guide policymakers, hospital administrators, and AI developers in creating or refining relevant regulatory frameworks, training programs, and technological safeguards hat align better with clinical realities and ethical standards. We do not claim generalizability beyond this setting; rather we illustrate how understanding AI at the frontline can foster ethically sound and patient-centered approaches. The findings may also reveal how or why certain AI models succeed in one local environment but not another, emphasizing the importance of context, data representativeness, and user trust.

Ultimately, by highlighting healthcare professionals’ local experiences and perspectives, this study offers a more comprehensive picture of AI’s everyday impact in clinical environments. While the literature has amply documented AI’s ability to improve efficiency and reduce costs, the ethical dimensions—especially those emerging from the direct interplay of clinicians and AI systems—remain inadequately understood. Bridging this gap is essential to ensure that AI’s integration in healthcare does not simply automate processes or cut expenses, but also respects the core tenets of patient welfare, equity, and accountability. In this way, our work contributes new insights on how best to shape AI strategies, policies, and designs to promote ethically sound and patient-centered outcomes.

## Methods

### Aim, design, and setting

The main aim of this study was to explore how healthcare professionals interpret, implement, and evaluate AI tools in clinical decision-making, focusing on ethical considerations such as accountability, transparency, and potential bias. Because this work was confined to a single NHS Trust context, we do not claim generalizability beyond that local setting. A qualitative design was adopted, grounded in phenomenological principles [10] that prioritize the subjective experiences and personal interpretations of participants. Phenomenology was deemed especially relevant given the multifaceted nature of AI integration in healthcare, where personal perceptions can reveal challenges, benefits, and ethical dilemmas not captured by purely quantitative measures. By emphasizing lived experiences, this approach allowed us to examine how participants understood and negotiated the opacity of AI systems, the sharing of responsibility for patient outcomes, and the potential for biased decision-making.

The study was conducted across multiple hospitals within one NHS Trust in the West Midlands, United Kingdom, each displaying varying levels of AI adoption. Some had integrated advanced imaging analytics into everyday practice, while others had more limited, pilot-stage AI initiatives. This range ensured that participants encompassed both early and later adopters, as well as those at different levels of enthusiasm or skepticism about AI-driven tools. We emphasize that our findings reflect the local experiences of staff at these sites and are not intended to be applied universally.

### Participant characteristics

We first used purposive sampling to capture a breadth of clinical roles, then applied snowball sampling to reach IT specialists and AI developers who were less visible in staff directories. Inclusion criteria required that individuals be employed within the hospital setting for at least one year, have direct or indirect exposure to AI-supported clinical systems, and voluntarily consent to participate. Exclusion criteria eliminated those without any exposure to AI or those unable to grant informed consent for any reason.

From these efforts, approximately 40 participants were recruited, comprising clinicians (such as doctors, nurses, and biomedical scientists), AI developers, IT specialists, and healthcare administrators. Fifteen participants identified as experienced clinicians with a history of working closely with AI-based tools, ten were AI experts or IT professionals involved in designing or maintaining AI systems, ten were administrators responsible for managing AI related activities, and five were clinicians who were relatively new to AI use. Out of the total 40 participants, 25 opted to participate in one-on-one interviews, while 15 took part in focus group discussions. This distribution ensured both a depth of individual reflections and the potential for interactive dialogue around shared AI-related challenges and successes.

### Demographics

In this qualitative study, 40 participants were recruited, comprising 28 clinicians, 6 AI developers, and 6 departmental administrators. Clinicians spanned various roles—clinical scientists, biomedical scientists, laboratory technicians, radiology specialists, nurses, and doctors—ensuring a wide spectrum of expertise and exposure to AI-driven tools. The decision to recruit 40 participants was guided by the principle of data saturation, whereby interviews continued until no new insights emerged. All participants were selected based on the direct or indirect influence of AI in their daily work. For instance, clinicians described using AI to streamline lab diagnostics, flag anomalies in patient imaging, or manage triage systems. AI developers refined algorithms, integrated them into electronic health records, and maintained predictive models for patient risk assessments, while departmental administrators oversaw the integration of AI into hospital workflows, focusing on policy compliance, staff training, and ethical considerations.

By including individuals across these diverse roles and real-world AI applications, the study captured a broad perspective on the integration, challenges, and ethical implications of AI in clinical decision-making. Table 1 provides an overview of the demographic characteristics, including gender, clinical experience, years of AI-assisted systems use, and specialty.

**Table 1:** Demographic characteristics (*n* = 40)

Characteristic	Item	Frequency	Percentage (%)
**Gender**	Male	22	55.0
	Female	18	45.0
**Clinical Experience**	Less than 5 years	6	15.0
	5–9 years	15	37.5
	10–14 years	10	25.0
	15 years or more	9	22.5
**Specialty (Clinicians Only**, ***n* = 28)**	Clinical Scientist	4	14.3
	Biomedical Scientist	8	28.6
	Laboratory Technician	5	17.9
	Radiology Specialist	3	10.7
	Nurse	2	7.1
	Doctor	6	21.4
**Years of Experience Using AI Assisted Systems**	Less than 1 year	10	25.0
	1–3 years	15	37.5
	4–6 years	8	20.0
	7 years or more	7	17.5
**Role**	Clinicians	28	70.0
	AI Developers	6	15.0
	Departmental Administrators	6	15.0

All participant quotations in this paper have been lightly edited for brevity and clarity. Minor grammatical refinements and the removal of extraneous filler words were made to ensure readability without compromising the substance or intent of the original remarks. To maintain transparency about these editorial choices, a selection of unedited, verbatim quotes is included in the appendix, allowing readers to observe participants’ spontaneous thinking and the ethical dilemmas they encountered—particularly around accountability, transparency, and bias in AI supported clinical decision-making.

### Data collection procedures

To capture the depth and breadth of participants’ experiences, semi-structured interviews were held with 25 participants, each session running for approximately 45 to 60 min. Some participants—15 in total—opted to join focus group discussions, each lasting around 60 min with groups of 5 to 6 people. In the individual interviews, participants often provided detailed, personal accounts of how AI affected their decision-making and ethical responsibilities; in contrast, the focus group format facilitated collective insights and sometimes revealed differing viewpoints about the same AI tools or processes. The interview guide was designed by the lead author in collaboration with the co-author, drawing on preliminary literature and pilot-tested with two senior clinicians. It covered topics such as perceived benefits of AI, potential workflow disruptions, issues of algorithmic opacity, and questions of accountability when AI-driven recommendations diverge from human clinical judgment.

All interviews and focus groups were audio-recorded after obtaining verbal consent from participants, with the recordings transcribed verbatim to create an accurate textual dataset. Transcripts were anonymized, with unique codes assigned to each participant, thereby removing references to personal identifiers, hospital names, or departmental specifics. Digital transcripts were securely stored on a password-protected computer system accessible only to the core study team.

No formal comparative interventions were introduced as part of this study. Rather, participants were encouraged to reflect on their existing experiences with any AI tools or processes present in their workplace, including both established systems and pilot-stage initiatives. While some hospitals were exploring AI to enhance diagnostic speed and accuracy, others were focusing on back-office operational tools, such as automated scheduling or real-time resource monitoring. These naturally occurring variations in AI use allowed for a wide scope of perspectives on ethical and practical hurdles.

### Data analysis

Data analysis followed the thematic analysis framework outlined by Braun and Clarke [24], which involves a structured, multi-phase process of coding, reviewing, and defining themes. Taking a deductive stance, we built an a-priori code book comprising five sensitising concepts: economic impact, efficiency, clinical impact, accountability & transparency, and bias derived from our study aims and the AI-ethics literature. Two researchers independently coded initial transcripts to generate an overarching codebook. Discrepancies were resolved through discussions designed to refine coding definitions, thereby ensuring consistency across the dataset. Once the codebook was deemed sufficiently stable, it was applied to the remaining transcripts. This iterative, cyclical process allowed for refinement of themes as new data emerged, enabling the study to capture multifaceted experiences of AI integration ranging from optimism about efficiency gains to concern over biases in training data or “black box” recommendations that lack explainability. While material relevant to all five sensitising concepts was identified, participants spoke most extensively about accountability, transparency, and bias; these three areas therefore receive particular emphasis in the Results.

Because some participants spoke in focus groups while others did so in private interviews, the analysis also considered the potential influence of group dynamics versus individual reflection. Focus group interactions sometimes triggered spontaneous debate or collective consensus on certain issues, whereas one-on-one interviews allowed for more personal, detailed narratives. Throughout the analysis, MAXQDA 24 software facilitated the systematic organization and retrieval of coded data. The emergent themes included questions about who bears responsibility for decisions in an AI-augmented environment, how transparency or opacity of AI outputs affects clinical trust and patient communication, and whether any known biases (such as underperformance in minority patient populations) had manifested in participants’ day-to-day practice. Data collection and analysis were undertaken concurrently, allowing the research team to adapt the interview guide as new focal areas, such as interpretability or user training, became increasingly salient in participants’ accounts.

### Ethical approval and considerations

Ethical approval for this study was granted by Aston University under its guidelines for non-invasive social research. The study involved interviews solely with healthcare professionals, and no identifiable patient data were collected, nor were any clinical interventions conducted. Additionally, approval from the Trust’s Caldicott Guardian was obtained to ensure adherence to national data confidentiality standards. All participants received an information sheet outlining the study’s aims, the voluntary nature of their involvement, and their right to withdraw at any time. Written informed consent was obtained from each participant prior to the interviews or focus groups. All data were anonymized, and no patient-related information was collected or stored. This study followed ethical guidelines to protect participants’ privacy and confidentiality, in line with the principles of the Declaration of Helsinki.

All recruitment and data collection procedures were carried out in coordination with local hospital administrators to avoid disruption to normal operations and to ensure fully voluntary staff participation. Participants received an information sheet outlining the study’s aims, the voluntary nature of involvement, the right to withdraw at any time, and the confidentiality measures protecting personal details. Written informed consent was obtained from each participant before any interview or focus group began. The participants were employees of the NHS Trust, and no patients were involved or patient data analyzed in the study. Interview transcripts were anonymized using unique participant codes. All electronic data were maintained on encrypted, password-protected systems, accessible only to the core research team. The resulting interviews and focus groups provided valuable insights into healthcare professionals’ perspectives on AI implementation and ethics, thereby advancing discussions on how to responsibly and effectively integrate AI-driven technologies into clinical practice.

## Results

### Overview

The findings of this study are split into three sections: (1) Accountability, (2) Transparency, and (3) Bias. These themes emerged from interviews and focus groups as pivotal to the ethical integration of AI into clinical decision-making. While participants acknowledged AI’s potential to streamline workflows and enhance diagnostic accuracy, they consistently returned to questions about who bears ultimate responsibility for AI-driven decisions, how explainable and interpretable AI systems need to be, and whether algorithms could inadvertently exacerbate existing disparities. In the sections that follow, we delve into each theme, highlighting participants’ perspectives, illustrative quotations, and the implications for healthcare practice.

### Theme 1: accountability in AI-Assisted decision making

Accountability in AI-assisted clinical settings elicited strongly held yet nuanced perspectives among clinicians, administrators, and AI developers. While most participants agreed that clinicians should ultimately bear responsibility for patient outcomes, many debated how far this accountability extends to the AI’s creators or the broader healthcare organization. Clinicians often viewed AI as a supportive tool yet warned that overreliance could blur lines of responsibility. One physician noted, *“I’m the one signing the treatment orders*,* but if the AI’s advice is wrong*,* how much of that blame falls on the developers?” (Doctor 4)*.

In the United Kingdom, AI adoption covers a broad spectrum of tools, from automated triage systems in emergency departments to machine learning algorithms for detecting subtle anomalies in radiological images. Participants praised these applications for speeding up diagnoses or spotting early indicators of sepsis and arrhythmias but stressed that final clinical decisions must remain in human hands. As a senior nurse explained, *“Even when the AI flags something serious*,* I want to verify with my own clinical judgment. Otherwise*,* it feels like I’m just blindly following an algorithm.” (Nurse 1)* Several clinicians also mentioned difficulties in justifying decisions based on opaque “black box” models, especially if subsequent outcomes contradicted the AI’s recommendations. A junior doctor using an NHS-approved imaging analysis AI for chest X-rays added, *“It’s fantastic at spotting subtle nodules*,* but if it recommends further invasive tests and turns out to be wrong*,* patients might blame me*,* not the system.” (Doctor 7)*.

Administrators generally mirrored these concerns, emphasizing human oversight through formal protocols requiring manual review of AI outputs—particularly when an algorithm deviates from expected norms. One administrator highlighted ongoing performance tracking:

*“We log every recommendation the AI makes and whether staff follow or override it*,* so if we see consistent errors*,* we investigate both the algorithm and user behavior.” (Administrator 3)*.

Such safeguards aim to preserve clinicians’ autonomy and patient safety, while capitalizing on AI’s efficiencies—for instance, predictive maintenance of hospital equipment or automated workforce scheduling. Administrators also believed hospital leadership and AI developers share responsibility for ensuring these tools meet accuracy standards and undergo routine audits.

Developers, for their part, stressed that they design assistive rather than autonomous solutions. Their systems often feature traceability measures—like monitoring logs, alert thresholds, and feedback loops—allowing clinicians to validate or challenge AI suggestions. A lead developer stated, *“We don’t want an AI model that just spits out a verdict. We build in a feedback loop so clinicians can question or confirm the AI’s suggestion*,* which helps trace accountability.” (Developer 2)* Yet formalizing liability for AI errors remained problematic, especially in complex, multi-layered applications like automated triage or real-time risk stratification. Another developer posed the question, *“If the system misprioritizes a patient’s acuity*,* is that a user error or an algorithmic fault? We need an agreed protocol for flagging these incidents.” (Developer 5)*.

Overall, 28 of the 40 participants (70.0%) believed clinicians should bear primary responsibility for patient outcomes, while 23 (57.5%) felt accountability should be partly shared with AI developers or the wider institution. Meanwhile, 20 participants (50.0%) cautioned against overreliance on AI or insisted that AI outputs be reviewed by human staff before action is taken. Table 2 summarizes these accountability perspectives. Although participants recognized how AI can streamline decision-making—be it for diagnostics, triage, or operational tasks—many insisted on retaining explicit clinician control. This emphasis on human oversight and system traceability suggests healthcare institutions need clear accountability frameworks to avert ambiguity if errors occur. Developers’ commitment to collaborative designs and administrators’ vigilance in performance tracking further demonstrate a collective effort to delineate responsibility in an era where AI is increasingly central to daily clinical practice.

**Table 2:** Views on accountability in AI-Assisted decision making (*n* = 40)

Accountability Theme	No. of Participants (%)
Clinicians should bear responsibility	28 (70.0)
Accountability should be shared between AI & clinicians	23 (57.5)
Concerns about overreliance on AI	20 (50.0)
AI recommendations must be reviewed by humans	20 (50.0)
AI systems must include checks and balances	17 (42.5)

### Theme 2: transparency in AI-Assisted decision making

Participants consistently cited transparency as crucial for trustworthy AI-driven care, especially in high-stakes or complex cases. Although UK-based AI tools—such as radiology image classifiers or risk stratification modules—often proved accurate, clinicians sometimes struggled to discern how these systems arrived at specific conclusions. One biomedical scientist recalled using CellaVision, an NHS-certified AI system widely adopted in hematology labs to assist with red blood cell morphology and white blood cell differentials: *“It flagged an odd pattern in the red blood cell morphology*,* suggesting early signs of a rare anemia. But the software gave no rationale for why those cells were abnormal. We had to investigate further manually*,* and it left us unsure how much to trust its recommendation.” (Clinician 6)*.

This lack of explainability was especially problematic in cases where clinicians or scientists required more detail to justify follow-up tests or treatments. A senior doctor noted, *“If I can’t see the logic behind an alert*,* how do I explain that to my patient?” (Doctor 5)* Administrators similarly acknowledged these difficulties, describing attempts to simplify AI outputs with color-coded summaries. One administrator remarked: *“We try to label AI findings clearly*,* so staff can act quickly. But if there’s no rationale*,* clinicians stay cautious.” (Administrator 4)*.

Despite these measures, participants recognized that deep learning methods can remain opaque, even when interpretability features like “heat maps” are added. One AI engineer working on automated triage systems said, *“We’re constantly balancing transparency with predictive power*,* and that’s tough for a complex neural network.” (Developer 2)* Clinicians generally welcomed partial solutions but stressed that greater clarity is necessary in high-risk or atypical scenarios. Administrators also introduced short tutorials on verifying AI suggestions or detecting false positives, aiming to minimize blind trust in “black box” algorithms.

Table 3 highlights the main transparency concerns. Participants most frequently cited a lack of detailed explanations for rare conditions and the inherent complexity of deep learning as obstacles to fully trusting AI outputs. While they appreciated AI’s ability to expedite diagnoses—whether in imaging or triage—many argued that transparency extends beyond merely providing results; it requires enabling clinicians to see, if only briefly, why the AI flagged an anomaly or recommended a certain action.

**Table 3:** Transparency concerns in AI-Assisted decision making (*n* = 40)

Transparency Concern	No. of Participants (%)
Lack of explanations for rare conditions	16 (66.7)
Transparency is better in routine cases	15 (62.5)
Need for simplified AI outputs	14 (58.3)
Deep learning models are hard to interpret	12 (50.0)
Existing strategies to enhance transparency	10 (41.7)

### Theme 3: Bias in AI and potential harm in AI algorithms

Bias in AI emerged as a persistent worry among clinicians, administrators, and developers, particularly for underrepresented patient groups and rare medical conditions. Participants noted that even popular systems in the UK—like automated triage or hematology image analyzers— could produce misleading outputs if training data were not diverse enough. One biomedical scientist stated: *“We discovered the AI under-reading certain cell abnormalities in minority populations*,* so we had to double-check everything. It really set off alarm bells.” (Clinician 11)*.

Such discrepancies can lead to delayed or incorrect diagnoses, a serious risk where timely intervention is crucial. A radiology specialist observed that AI might excel at detecting common pathologies but fail to address unusual cases: *“It catches typical tumors*,* but we see it missing rare or atypical ones—likely because the model never saw enough examples in training.” (Doctor 9)*.

Administrators and developers were aware of these challenges, outlining bias testing, performance audits, and retraining with more representative datasets as key mitigation strategies. One developer recalled correcting a system that initially favoured elderly patients: *“We saw a skew*,* so we retrained on balanced data. Accuracy improved*,* but bias can creep back if we don’t keep updating the dataset.” (Developer 4)*.

Still, many clinicians remained cautious, stressing that narrow training sets can perpetuate existing disparities or obscure clinically significant variations. Administrators agreed that continuous auditing is essential as healthcare demographics evolve and new data emerge. They also pointed out that transparency (discussed earlier) is crucial for detecting bias, since an opaque system can conceal skewed decision rules until harm occurs.

Table 4 summarizes the primary bias concerns. Clinicians most frequently cited misleading recommendations for minority groups and underperformance in diagnosing rarer conditions, while administrators and developers underscored diverse training data and regular audits as vital. These findings suggest that, in our local context, maintaining equity in AI-driven healthcare demands proactive strategies—from selecting balanced datasets to establishing robust feedback loops that capture real-world performance across varied patient populations.

**Table 4:** Bias issues and mitigation measures in AI systems (*n* = 40)

Bias Concern / Measure	No. of Participants (%)
Misleading recommendations for minority groups	16 (66.7)
Manual review of AI outputs for certain patients	14 (58.3)
AI underperformance in diagnosing rare conditions	12 (50.0)
Need for more diverse training data	15 (62.5)
Regular bias testing and audits implemented	12 (50.0)

Ultimately, participants viewed bias as a serious threat to both patient safety and fairness, warning that insufficiently diverse data or unmonitored algorithmic drift could undermine the accountability and transparency crucial for ethical AI use. Even seemingly high-performing systems risk introducing inequities if their design and deployment overlook population-level nuances. As the preceding themes imply, addressing bias in AI requires continuous vigilance, and a willingness to refine tools and protocols whenever new evidence of skew emerges.

### Conclusion of results

Overall, participants recognized AI’s capacity to streamline diagnostic workflows and improve clinical outcomes in their local NHS environment but cautioned that its ethical and practical success hinges on three factors. Accountability demands that clinicians retain ultimate responsibility while developers and institutions provide safe, assistive solutions. Transparency is essential so clinicians can interpret or question AI outputs, rather than blindly accepting “black box” recommendations. Bias remains a persistent concern, particularly for minority groups and rare conditions, underscoring the need for diverse training data and continuous audits. Although these findings reflect staff experiences within a single NHS Trust and are not intended for generalization beyond that context, addressing these themes collaboratively lays the groundwork for a responsible, patient-centered future of AI-assisted healthcare.

## Discussion and conclusion

### Discussion

This study demonstrates that AI integration in clinical decision-making offers considerable advantages, particularly in boosting diagnostic accuracy, expediting clinical workflows, and reducing healthcare expenditures by optimizing resource use. Participants highlighted the ability of AI systems to minimize idle time and streamline processes, echoing prior research indicating that AI can diminish manual labor and human error, ultimately leading to cost savings [5, 25]. Some interviewees acknowledged the significant up-front costs of AI adoption [26], though they remained optimistic that these investments could be offset by long-term gains [13].

Beyond economic considerations, participants consistently returned to three interrelated themes critical to the ethical deployment of AI: Accountability, Transparency, and Bias. Their discussions align with the literature’s warning that while AI can streamline diagnostics and reduce clinician workload [27], substantial ethical and procedural complexities must be addressed. Clinicians underscored the importance of human oversight, expressing reservations over “black box” systems that complicate the delineation of responsibility, as some argued that accountability should be shared with AI developers. This tension echoes concerns about liability and explainability in AI-reliant environments [5, 7].

While accountability, transparency, and bias are well-known in AI ethics, our data revealed three NHS-specific twists. Clinicians framed accountability through the Trust’s Serious Incident Framework, expressing concern that AI audit logs are not integrated into that process. Transparency worries centered on whether AI audit trails can be ingested by the NHS Spine, hindering retrospective case reviews. Staff also highlighted dialect bias in voice-triage tools, noting that patients with strong regional accents are under-flagged—an issue seldom documented outside the UK. These context-bound insights extend current debates and suggest governance levers unique to NHS settings.

### Ethical Trade-offs: accuracy versus interpretability

Underpinning these accountability and transparency dilemmas is a deeper conflict between highly accurate AI models and the need for interpretability. Many participants recognized that complex algorithms, particularly deep neural networks, can outperform simpler, more transparent models in tasks like anomaly detection. However, reliance on such “black box” systems raises questions of how clinicians can justify treatment decisions to patients or defend them in medico-legal contexts if they themselves do not fully understand the AI’s reasoning process. This finding reflects the broader debate in AI ethics [28]: whether predictive performance should take precedence over explainability, or whether there should be an ethical imperative to preserve a degree of interpretability—even if it comes at the cost of some accuracy.

Participants’ concerns thus highlight the ethical trade-off at the heart of AI integration, suggesting that neither extreme—a purely explainable but less accurate system, nor a highly accurate but opaque model—is fully satisfactory without appropriate safeguards and guidelines.

Closely tied to these accountability dilemmas was the pervasive emphasis on Transparency. Participants’ experiences confirmed that opaque AI outputs, despite high accuracy, can undermine user trust and impede clinical justification, particularly in the case of rare conditions. These worries mirror Challen et al.’s call for frequent updates and audits to maintain integrity and adapt AI systems to evolving clinical contexts [29]. Similarly, Fazakarley et al. emphasized that transparency is pivotal in avoiding disparities in care [30], a point supported by clinicians who stressed the need for interpretable AI models that allow them to grasp why an alert or recommendation is triggered.

### Bias and its consequences

Participants also spoke at length about Bias, asserting that AI systems trained on narrow datasets risk magnifying health inequities among minority ethnic groups or less common conditions [9, 31]. Their cautionary stance resonates with the recognition that bias emerges when algorithms learn from non-representative or incomplete data, leading to missed diagnoses and late interventions, especially for underrepresented populations. While the adoption of diverse training samples and audits can partially correct these shortcomings, ongoing vigilance is crucial. Participants advocated both continuous retraining of AI on more inclusive data and clearer oversight protocols to catch and address emergent biases before they harm patient outcomes.

Taken together, these findings point to a complicated yet hopeful picture within this NHS Trust. AI holds tangible potential to enhance patient care efficiency and accuracy, yet that promise can be undercut by accountability gaps, opaque decision-making processes, and algorithmic biases. Ensuring that AI truly augments rather than complicates clinical work requires sustained collaboration among healthcare professionals, AI developers, administrators, and policymakers.

### Regulatory and governance measures

Participants also highlighted the need for robust regulatory and governance frameworks to provide clearer guidelines on where liability lies, how transparency should be maintained, and how biases should be detected and mitigated. While some pointed out existing regulatory mechanisms within the NHS, they nonetheless argued that bodies such as the General Medical Council (GMC) in the UK—or equivalent authorities elsewhere—could further clarify the professional and legal responsibilities associated with AI-driven recommendations. Specifically, participants asked whether more frequent audits should be mandatory, or whether there should be a standardized “explainability requirement” for certain high-risk applications. These views reflect a growing consensus in the literature that regulatory oversight must evolve in parallel with AI’s technological sophistication, ensuring that ethical standards keep pace with the rapid deployment of AI in healthcare.

### Recommendations

Addressing these themes calls for a multifaceted approach. First, healthcare professionals must remain central to AI adoption, ensuring that the technology is clinically relevant and ethically sound. AI introduction should be carefully assessed for cost-effectiveness, with institutions conducting regular evaluations to verify that initial investments are recouped through operational benefits. As part of this oversight, it is essential to institute frameworks that assign clear liability and maintain human oversight, preventing clinicians from becoming mere implementers of AI suggestions without fully comprehending their rationale.

Another consideration is that healthcare providers should receive ongoing training and support on AI’s capabilities and limitations, including potential bias or lack of transparency. Regular feedback loops between clinicians and AI developers would help refine AI tools in real time. Meanwhile, regulatory bodies should craft guidelines that require diverse training datasets, explainability features for high-stakes decisions, and continuous performance audits—thus aligning AI usage with ethical standards and mitigating disparities among vulnerable groups.

### Limitations

One principal limitation of this study is the exclusion of patient respondents, a choice made to protect patient confidentiality. This omission restricts insight into how AI-assisted care affects patient satisfaction, autonomy, and trust in clinical outcomes. While participants speculated about patient attitudes—some believing patients might share concerns about opaque algorithms, others suggesting quick diagnoses might be prioritized over transparency—these perspectives were not empirically investigated. Future research should directly involve patients through interviews or surveys to ascertain their views on accountability, transparency, and potential biases in AI systems. Such patient-centered research would inform more nuanced policy guidelines and ensure that AI solutions align with public expectations as well as clinical feasibility.

Second, participants discussed a heterogeneous mix of AI applications—approximately two-thirds referenced diagnostic tools while one-third referred to operational tools (e.g., bed-management dashboards). Perceptions of transparency and bias may therefore vary by application type, and our findings may not apply equally across different categories of AI systems. Third, of the 58 staff invited, 40 participated (69% uptake), raising the possibility of self-selection bias among those with particularly strong views on AI. Those who declined participation may have held different perspectives on AI implementation, potentially limiting the representativeness of our findings. Fourth, focus-group dynamics may have muted dissent, especially when junior and senior staff participated together. While we attempted to mitigate this through triangulation with 25 one-to-one interviews, power-gradient effects may persist and could have influenced the expression of critical views about AI systems.

A further constraint arises from the single-site scope of the study, limiting the generalizability of results. Although participants offered a broad range of professional viewpoints, additional research involving multiple institutions or larger, more diverse samples could confirm whether these themes hold across varied clinical contexts. Similarly, the qualitative design, while yielding rich accounts, does not permit quantification of AI’s broader impact on operational efficiency or ethical issues. Moreover, the study did not systematically examine how well clinicians are trained to operate AI systems, beyond anecdotal references to training gaps. Future research may explore whether structured curricula or ongoing learning sessions enhance AI adoption and usability. Lastly, the feedback channels between front-line users and AI developers received only passing mention, suggesting an area for further exploration in subsequent studies.

### Future studies

Future investigations might involve directly surveying or interviewing patients to capture their perceptions of AI-assisted diagnosis and treatment, potentially revealing new angles on accountability, transparency, or bias. Additional single-or multi-site studies could also measure the efficacy of structured clinician training sessions in improving AI adoption rates, reducing errors, and fostering trust. Longitudinal research might reveal how AI tools evolve as they are recalibrated with fresh data or deployed in different clinical settings, and whether accountability, transparency, and bias issues shift in tandem. Additionally, more quantitative research—perhaps comparing patient outcomes or measuring cost savings from AI systems across various specialties—would complement these qualitative insights.

### Conclusion

This study highlights AI’s considerable promise for enhancing diagnostic accuracy, reducing clinical workloads, and improving efficiency, while also underscoring the ethical challenges that arise when AI is deeply integrated into clinical decision-making. Participants repeatedly returned to three key issues—Accountability, Transparency, and Bias—as being critical for ethical, effective AI adoption. Balancing these dimensions requires clear accountability frameworks that preserve clinicians’ oversight, transparent models that allow for at least some interpretability, and diverse, continuously audited datasets that reduce the risk of harmful recommendations. As AI technology continues to expand, collaboration among clinicians, administrators, developers, and policymakers will be crucial for establishing guidelines that reflect both technical and ethical realities. By addressing accountability, transparency, and bias with ongoing diligence, healthcare systems can harness AI’s potential while maintaining the trust, equity, and integrity that lie at the heart of patient-centered care.

## Electronic supplementary material

Below is the link to the electronic supplementary material.

Supplementary Material 1

## Data Availability

The qualitative datasets (interview transcripts, focus group data) generated and analyzed during the current study are not publicly available to protect participant confidentiality. However, de-identified versions of the transcripts may be made available from the corresponding author upon reasonable request and with appropriate institutional approvals.

## Abbreviations

AI: Artificial Intelligence
DL: Deep Learning
EHR: Electronic Health Record
ML: Machine Learning
NHS: National Health Service
MAXQDA: (Name of the data analysis software)
