# Supplementary material for: Evaluating accountability, transparency, and bias in AI-assisted healthcare decision- making: a qualitative study of healthcare professionals’ perspectives in the UK
Source: BMC Med Ethics. 2025 Jul 8;26:89. doi: 10.1186/s12910-025-01243-z (PMC12235780; doi:10.1186/s12910-025-01243-z)
Supplement: Supplementary file 1 — Supplementary Material 1 [file 12910_2025_1243_MOESM1_ESM.pdf]

Appendix A: Interview Questions

I. Clinicians with Established AI Experience

1. **Accountability**
    - When an AI tool suggests a clinical decision or diagnosis, how do you see the division of responsibility if something goes wrong (e.g., adverse outcomes)?
    - Do you feel AI developers, clinicians, or healthcare institutions should share accountability? Why or why not?
2. **Transparency**
    - How clearly do you understand the reasoning behind AI outputs (e.g., flagged abnormalities, risk scores)?
    - If explanations are lacking, how does this affect your trust in the AI system and your decision-making process?
3. **Bias**
    - Have you observed any AI results that seemed biased or systematically less accurate for certain patient groups (e.g., ethnic minorities, rare conditions)?
    - How did you detect and address these biases, and what additional measures could help in future?
4. **Balancing Clinical Judgment and AI**
    - How do you integrate AI's recommendations with your own clinical expertise?
    - Have you ever had to override AI-generated guidance, and if so, what factors informed your decision?
5. **Training and Support**
    - Did your initial AI training discuss issues like accountability, transparency, and bias?
    - What further training or guidelines would help you address these ethical challenges?

II. AI Experts / Developers / IT Professionals

1. **Accountability Frameworks**
    - From a design standpoint, how do you envision accountability for AI-generated decisions?
    - What mechanisms exist (e.g., audit trails) to trace errors back to either model design or user handling?
2. **Transparency Methods**
    - What strategies or tools do you use to make AI outputs interpretable for clinical staff (e.g., explanations, visual overlays)?
    - What are the primary barriers to achieving greater transparency, especially with deep learning models?
3. **Bias Detection and Mitigation**
    - How do you test for potential biases in the datasets you use for training (e.g., underrepresentation of certain demographics)?

- Can you share an example where you identified and corrected bias in an AI model?

4. **Collaboration with Clinicians**
    - How do clinicians typically report issues or concerns related to bias or unclear AI outputs, and what is your process for addressing these?
    - What feedback loops or update cycles do you have to continuously refine the AI model?
5. **Responsibility for Deployment**
    - In your view, where does your role end and the healthcare institution's role begin regarding safe AI deployment?
    - Do you think developers should have legal or ethical liability if an AI tool contributes to misdiagnosis or patient harm?

III. Healthcare Leaders / Administrators

1. **Policies on Accountability**
    - What policies or frameworks does your organization have in place to define who is accountable if AI-driven decisions lead to errors or patient harm?
    - How do you communicate these policies to both clinical staff and AI developers?
2. **Transparency and Regulatory Compliance**
    - Are there guidelines or standards (e.g., from NHS or professional bodies) that mandate transparency for AI tools used in your facility?
    - How do you ensure these guidelines are followed in day-to-day clinical practice?
3. **Monitoring and Auditing Bias**
    - What processes or committees exist within your organization to audit AI systems regularly for bias or disparate outcomes?
    - Have you seen any instances where specific patient demographics experienced systematically different results?
4. **Risk Management and Safeguards**
    - How do you handle incidents or "near misses" that appear tied to AI recommendations?
    - What risk mitigation strategies (e.g., second-check protocols, mandatory overrides) are in place?
5. **Strategic Outlook**
    - Considering accountability, transparency, and bias concerns, how do you see AI expanding in your organization over the next few years?
    - Do these concerns limit adoption, or have you found ways to address them while still pushing for innovation?

IV. Clinicians New to AI

1. **Initial Impressions of Accountability**
    - With limited exposure to AI, do you feel clear about who is responsible if something goes wrong based on an AI recommendation?
    - Have you received any specific guidance from your institution on these accountability questions?
2. **Transparency and User Confidence**
    - How well do you understand the rationale behind the AI outputs you've encountered so far?
    - Does the AI system offer explanations or visual indicators, and does that influence your confidence?
3. **Detecting Bias Early**
    - Have you noticed or suspected that the AI might be less accurate for certain patients or conditions?
    - What would help you detect or escalate potential bias issues if you come across them?
4. **Learning and Adaptation**
    - Does your current training program address topics like bias, accountability, or transparency?
    - What additional resources or mentoring would help you integrate AI more effectively into your practice?
5. **Future Steps**
    - Are there specific safeguards or adjustments you would recommend—based on your early experiences—to better handle accountability, transparency, and bias?
    - How might these improvements change your day-to-day reliance on AI?
